# Chronic obstructive pulmonary disease burden attributable to tobacco and the trend change from 1990 to 2021 in China

**DOI:** 10.18332/tid/200196

**Published:** 2025-02-10

**Authors:** Zhenhong Zhang, Kai Wang, Guoxiang Xu, Rumin Zhang

**Affiliations:** 1 School of Clinical Medicine, Shandong Second Medical University, Weifang, People’s Republic of China; 2 Department of Critical Care Medicine, Zibo Central Hospital, Zibo, People’s Republic of China; 3 School of Clinical Medicine, Binzhou Medical University, Binzhou, People’s Republic of China

**Keywords:** China, chronic obstructive pulmonary disease, Global Burden of Disease

## Abstract

**INTRODUCTION:**

Chronic obstructive pulmonary disease (COPD) poses a serious disease burden in China, and tobacco control is considered an effective and feasible means to reduce this burden. This study analyzes the impact of tobacco on the burden of COPD in China from 1990 to 2021.

**METHODS:**

This study conducted a comprehensive secondary dataset analysis of COPD attributable to tobacco in China using data from the Global Burden of Disease (GBD) database. This covers a range of statistics, including number of deaths, mortality rates, disability-adjusted life years (DALYs), and DALY rates. Using Joinpoint regression analysis methods, we calculated the annual percentage change (APC) and average annual percentage change (AAPC) to assess trends in COPD attributable to tobacco for various age groups and gender in China.

**RESULTS:**

In 2021, the age-standardized mortality rate (ASMR) of COPD attributable to tobacco in China was 35.46 per 100000, and the age-standardized DALY rate (ASDR) was 589.75 per 100000, both of which were higher than global levels. In China, the ASMR attributable to tobacco showed a consistent decline from 1990 to 2021 (AAPC= -3.69%, p<0.001), as did the ASDR (AAPC= -3.73%, p<0.001), consistent with trends observed globally and across five SDI regions, with China experiencing the fastest decline. In both 1990 and 2021, the burden of COPD attributable to tobacco was greater in males compared to females. During the years from 1990 to 2021, the ASMR of COPD attributable to tobacco and the ASDR showed a declining trend in males (AAPC= -3.29% and -3.41%, respectively) and in females (AAPC= -4.99% and -4.62%, respectively) (all p<0.001). The impact of COPD linked to tobacco use in China increased with age from 1990 to 2021, with the highest mortality and DALY rates observed in the population aged ≥70 years. Regarding secondhand smoke exposure, ASMR for females was 6.29 per 100000 and the ASDR was 119.03 per 100000, while the corresponding values for males were 7.80 per 100000 and 113.10 per 100000 , indicating a higher burden among females.

**CONCLUSIONS:**

From 1990 to 2021, the age-standardized mortality rate and age-standardized DALY rate of COPD attributable to tobacco in China showed a declining trend; however, there remains a slight gap compared to global levels. Significant differences in smoking exposure were observed based on gender and age, with a heavier burden among males and the elderly.

## INTRODUCTION

Chronic obstructive pulmonary disease (COPD) is a common and serious chronic respiratory disease characterized by chronic and progressive airflow limitation, which may be partially reversible in some cases, significantly impacting an individual’s quality of life^1^. The global rates of incidence and mortality associated with COPD are alarmingly elevated, particularly in developing countries, resulting in substantial economic and social burdens. By 2030, COPD is expected to rank as the third leading cause of death globally, with >4.5 million people succumbing to this disease or related conditions each year, highlighting its significance as a major public health challenge on a global scale^2,3^. In China, COPD is a leading cause of death in both urban and rural areas, with a higher prevalence in rural regions, resulting in a greater disease burden than in developed countries^4^. Research indicates that while the disease burden of COPD on the Chinese population has shown a general downward trend in recent years, it still remains at a relatively high level^5^.

Since the implementation of reform and opening up policies, China has experienced rapid economic development, leading to smoking becoming a lifestyle choice for certain groups, which has contributed to persistently high smoking rates in the country^6^. Cigarette smoke significantly contributes to the onset and progression of various diseases, as the oxidative stress it generates results in lung damage and cancer^7^. Numerous studies have confirmed that smoking is a primary risk factor for COPD and acknowledged as an independent factor that triggers and worsens its progression^8^. Smoking-induced apoptosis of pulmonary vascular endothelial cells is critical in initiating and contributing to the pathogenesis of COPD, solidifying smoking as a primary risk factor for this disease^9^.

Despite the wealth of related studies, research on the disease burden of tobacco-related COPD in developing countries like China remains relatively scarce. Given that the disease burden of COPD has emerged as a serious public health issue, this study aims to investigate the impact of smoking on the burden of COPD in China. Using data from the 2021 Global Burden of Disease Study (GBD), the research analyzes the effects of tobacco – encompassing both active smoking and secondhand smoke exposure – on COPD across various age groups and genders from 1990 to 2021.

## METHODS

### Research design and data acquisition

This research is a secondary dataset analysis of the GBD data and does not include animal or human participants; consequently, informed consent and ethical approval were not necessary. At the forefront of research, the GBD project, organized by the Institute for Health Metrics and Evaluation at the University of Washington in the USA, undertakes the collection, incorporation, and analysis of data related to 88 risk factors spanning 204 countries and 811 regions. The project regularly publishes comprehensive statistics on incidence, prevalence, and disability-adjusted life expectancy, categorized by country, year, gender, cause, and age^10^. The GBD database provides a level of information on diseases, risk factors, deaths, and disabilities associated with health burdens caused by diseases, establishing it as the foremost resource for understanding the global burden of disease. This study centered on COPD as the main disease of focus. The definition of COPD comes from the Global Initiative for Chronic Obstructive Lung Disease (GOLD) classification: after bronchodilator treatment, the ratio of FEV1/FVC (forced expiratory volume in one second/total forced expiratory volume) in pulmonary function tests is <0.7. ICD-10 codes associated with COPD include J41, J42, J43, J44, and J47^11^. We extracted data on the tobacco-attributable burden of COPD from 1990 to 2021 for China, as well as on a global scale and among different Socio-Demographic Index (SDI) regions. These data include mortality rates, the number of deaths, DALY rates, all categorized by year, gender, and age. Here, we group ages into three categories: 15–49, 50–69, and ≥70 years. For the purpose of our study, smokers are defined as individuals who currently engage in daily or occasional use of any tobacco smoking products, including those who have used these products in the past. We include all forms of tobacco consumption, such as cigarettes, kretek, pipes, shisha, cigars, bidis, and other locally consumed smoking products. However, smokeless tobacco, e-cigarettes (commonly referred to as vaping products), and heated tobacco products are excluded from this definition^12^.

### Socio-Demographic Index (SDI)

The SDI is a composite measure that assesses a country’s level of development based on individual income, mean years of schooling, and total fertility rate. It categorizes countries into five groups: low, low-middle, middle, high-middle, and high SDI regions^10,13^. The SDI serves as a crucial indicator for evaluating the progress of a country or region in terms of development. The SDI ranges from 0 to 1, where higher values indicate a higher level of socio-economic development. A score of 1 indicates the utmost level of development, whereas a score of 0 indicates the minimum level^13^.

### Statistical analysis

This study utilized R Studio software (version 4.4.0) for data processing. The key indicators analyzed include the number of deaths, mortality rates, DALYs, DALY rates, and their 30-year trend related to the burden of COPD caused by tobacco use. Using Joinpoint software, we calculated the annual percent change (APC) and average annual percent change (AAPC), as well as their 95% confidence intervals (95% CI), for the standardized mortality rate and standardized DALY rate attributable to tobacco in China from 1990 to 2021. This analysis employs a logarithmic linear model within Joinpoint regression, and the default modeling strategy utilized was the Grid Search Method (GSM), enhanced by Monte Carlo permutation techniques for selecting the best model. The Joinpoint regression analysis methods yields the APC and AAPC for the duration of the study, including their respective 95% confidence intervals, with a significance level set at α=0.05^14^. This statistical method analyzes the trends of disease incidence over time. The model first fits the original data with the minimum number of connection points (for example, zero connection points represent a straight line-indicating that the overall trend of the data set is either monotonically decreasing or increasing). It then tests whether additional connection points are statistically significant and should be added to the model. The maximum number of connection points is related to the input data. Here, APC reflects the trend of changes in disease burden within a specific time period, while AAPC reflects the overall trend of changes in disease burden over the entire analysis period^15^. APC is calculated by comparing the changes between two time points, specifically by taking the number of deaths at the second time point, subtracting the number of deaths at the first time point, dividing by the number of deaths at the first time point, and finally multiplying by 100. This analysis aims to evaluate the time-related trends in tobacco consumption related to COPD and to identify significant annual changes. An APC>0 indicates an increase in the indicator during that segment, while an APC<0 signifies a decrease. Similarly, if the AAPC>0, the indicator is increasing year after year, and decreasing if <0. Furthermore, if the 95% CI for either APC or AAPC encompasses 0, this indicates that the change in the trend is not statistically significant^16^.

## RESULTS

### Burden of COPD attributable to tobacco from 1990 to 2021

**Figure 1:**
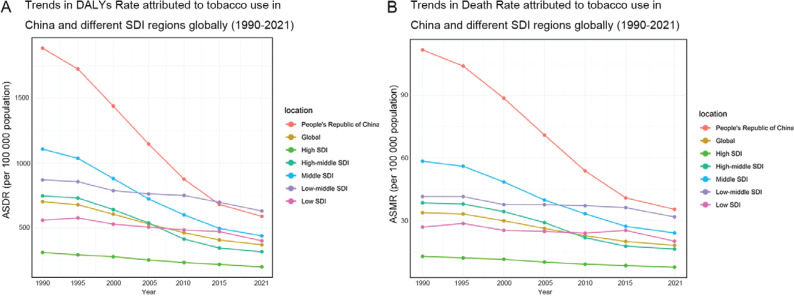
Age-standardized DALY rate and mortality of COPD attributed to tobacco in China, globally, and across SDI regions (1990–2021): A) Trends in DALY rates attributable to tobacco use in China and different SDI regions globally; B) Trends in death rate attributable to tobacco use in China and different SDI regions globally

From 1990 to 2021, the burden of COPD attributed to tobacco exhibited consistent trends in China, globally, and across five categories of SDI regions. Both the ASMR and ASDR demonstrated an overall downward trend. Notably, China experienced the fastest decrease, indicated by an AAPC of -3.69 for ASMR and -3.73 for ASDR. From 1990 to 2021, the ASMR and ASDR for COPD attributed to tobacco in China were consistently higher than the global averages and those of the five SDI regions. However, beginning in 2015, China’s ASDR fell below those of the low and middle SDI regions, although they continued to exceed the global level. In 2021, per 100000 individuals, the ASMR was 35.46 and the ASDR was 589.75, These numbers remain significantly above the global levels of 18.26 and 370.64 (Figure 1 and Table 1).

**Table 1:** Disease burden and the trends of COPD attributable to tobacco in China, 1990–2021

*Category*	*Indicator*	*Time*	*Percent change* *(95% CI)*	*p*
**ASMR**	APC	1990–1994	-1.07 (-1.83 – -0.29)	0.01
		1994–2004	-3.36 (-3.57 – -3.15)	<0.001
		2004–2007	-8.27 (-10.38 – -6.11)	<0.001
		2007-2010	-3.04 (-5.21 – -0.83)	0.011
		2010–2015	-5.47 (-6.17 – -4.75)	<0.001
		2015–2021	-2.44 (-2.89 – -1.99)	<0.001
	AAPC	1990–2021	-3.69 (-4.02 – -3.36)	<0.001
**ASDR**	APC	1990–1994	-1.47 (-2.11 – -0.82)	<0.001
		1994–2004	-3.68 (-3.86 – -3.50)	<0.001
		2004–2007	-7.77 (-9.58 – -5.91)	<0.001
		2007–2010	-3.14 (-4.99 – -1.25)	0.003
		2010–2015	-5.07 (-5.68 – -4.45)	<0.001
		2015–2021	-2.43 (-2.81 – -2.05)	<0.001
	AAPC	1990–2021	-3.73 (-4.01 – -3.45)	<0.001

ASMR: age-standardized mortality rate per 100000 population. ASDR: age-standardized disability-adjusted life year rate per 100000 population. APC: annual percentage change. AAPC: average annual percentage change.

### Burden of COPD attributed to tobacco in China from 1990 to 2021

From 1990 to 2021, the ASMR and ASDR for COPD related to tobacco in China decreased from 111.74 and 1884.73 in 1990 to 35.46 and 589.75 in 2021, all per 100000, respectively. These represent declines of 68.27% and 68.71%. The AAPC was -3.69% (95% CI: -4.02 – -3.36) for ASMR and -3.73% (95% CI: -4.01 – -3.45) for ASDR. Both trends were statistically significant (Table 1).

From 1990 to 2021, the trends in the ASMR and ASDR of COPD attributed to tobacco in China exhibited similar patterns, both showing a fluctuating decline. Notably, from 2004 to 2007, the reductions in the ASMR and ASDR attributed to tobacco were the most significant, with APC of -8.27% (95% CI: -10.38 – -6.11) and -7.77% (95% CI: -9.58 – -5.91), respectively (Table 1). Correspondingly, from 2004 to 2007, the ASMR decreased from 75.73 to 59.24, while the ASDR decreased from 1220.52 to 965.87 (Table 2).

**Table 2:** Disease burden and the trends of COPD attributable to tobacco in the Chinese population for different years, 1990–2021

*Year*	*ASMR*			*ASDR*		
	*All*	*Male*	*Female*	*All*	*Male*	*Female*
1990	111.74	193.55	59.65	1884.73	3117.39	967.21
1994	106.52	181.54	58.30	1771.03	2880.27	938.18
2004	75.73	142.70	34.83	1220.52	2139.35	557.36
2007	59.24	115.33	25.56	965.87	1727.09	421.32
2010	53.87	105.98	21.42	875.92	1585.97	357.03
2015	40.97	82.18	14.29	680.78	1246.92	257.68
2021	35.46	70.91	12.50	589.75	1079.53	225.13
**AAPC**	-3.69	-3.29	-4.99	-3.73	-3.41	-4.62

ASMR: age-standardized mortality rate per 100000 population. ASDR: age-standardized disability-adjusted life year rate per 100000 population. AAPC: average annual percentage change.

### Burden of COPD attributed to tobacco among different gender populations in China from 1990 to 2021

From 1990 to 2021, the ASMR and ASDR attributed to tobacco-related COPD among Chinese men were both higher than those among women, with a declining trend observed in both cases. The ASMR for tobacco-related COPD in Chinese men declined from 193.55 per 100000 in 1990 to 70.91 per 100000 in 2021, with an AAPC of -3.29% (95% CI: -3.69 – -2.88). The observed decline is statistically significant (p<0.001). Similarly, the age-standardized DALY rate for tobacco-related COPD in men fell from 3117.39 per 100000 in 1990 to 1079.53 per 100000 in 2021, with an AAPC of -3.41% (95% CI: -3.76 – -3.05, p<0.001).

From 1990 to 2021, ASMR and ASDR attributed to tobacco-related COPD among Chinese women decreased significantly. The ASMR fell from 59.65 per 100000 in 1990 to 12.50 per 100000 in 2021, with AAPC of -4.99% (95% CI: -5.45 – -4.52). The ASDR also declined from 967.21 per 100000 in 1990 to 225.13 per 100000 in 2021, with an AAPC of -4.62% (95% CI: -4.87 – -4.37). Both declining trends are statistically significant (p<0.001) (Table 3).

**Table 3:** Disease burden and the trends of COPD attributable to tobacco in Chinese males and females, 1990–2021

*Gender*	*ASMR*				*ASDR*			
	*1990*	*2021*	*AAPC (95% CI)*	*p*	*1990*	*2021*	*AAPC (95% CI)*	*p*
Male	193.55	70.91	-3.29 (-3.69 – -2.88)	<0.001	3117.39	1079.53	-3.41 (-3.76 – -3.05)	<0.001
Female	59.65	12.50	-4.99 (-5.45 – -4.52)	<0.001	967.21	225.13	-4.62 (-4.87 – -4.37)	<0.001
All	111.74	35.46	-3.69 (-4.02 – -3.36)	<0.001	1884.73	589.75	-3.73 (-4.01 – -3.45)	<0.001

ASMR: age-standardized mortality rate per 100000 population. ASDR: age-standardized disability-adjusted life year rate per 100000 population. AAPC: average annual percentage change.

### Burden of COPD attributed to tobacco among different age groups in China from 1990 to 2021

From 1990 to 2021, the mortality and DALY rates for COPD attributed to tobacco in China exhibited an increasing trend with age, peaking among individuals aged ≥70 years. In 1990, the mortality and DALY rates for individuals aged 15–49 years were 2.05 and 114.72, per 100000, respectively; for 50–69 years, 116.34 and 3545.32; and for ≥70 years, 1137.10 and 17997.40. By 2021, the mortality and DALY rates had declined significantly, with the age group 15–49 years showing rates of 0.69 and 52.80; 50–69 years, 24.79 and 873.59; and ≥70 years reaching 450.75 and 6639.64. From 1990 to 2021, the changes in mortality and DALY rates of COPD attributed to tobacco in different age groups in China showed a declining trend across all age groups, with the AAPC<0, and the results were statistically significant (Table 4). In addition, in any given year, the number of tobacco-related deaths was significantly age-related, increasing with age and peaking among individuals aged ≥70 years.

**Table 4:** Disease burden and the trends of COPD attributable to tobacco in the Chinese population for different age groups, 1990–2021

*Age (years)*	*Mortality rate (per 100000)*				*DALY rate (per 100000)*			
	*1990*	*2021*	*AAPC (95% CI)*	*p*	*1990*	*2021*	*AAPC (95% CI)*	*p*
15–49	2.05	0.70	-3.52 (-3.83 – -3.21)	<0.001	114.72	52.80	-2.48 (-2.73 – -2.24)	<0.001
50–69	116.34	24.79	-4.92 (-5.24 – -4.60)	<0.001	3545.32	873.59	-4.48 (-4.75 – -4.20)	<0.001
≥70	1137.10	450.75	-2.98 (-3.29 – -2.67)	<0.001	17997.40	6639.64	-3.20 (-3.47 – -2.93)	<0.001

AAPC: average annual percentage change.

### Comparative analysis of the burden of COPD caused by active smoking and secondhand smoke

In 2021, the disease burden resulting from active smoking was more significant among males than among females. Specifically, the number of male deaths was 492369 (ASMR 67.62 per 100000), while the number of female deaths was 70798 with ASMR of 10.19 per 100000. Additionally, the male ASDR was 1222.60, while for females it was 185.07. Among male residents, 65.44% of COPD deaths are attributable to active smoking, while the proportion is 13.24% among females. It is worth noting that for indicators related to secondhand smoke, female data are similar to, and slightly higher than, male data. There were 53328 male deaths and 64060 female deaths, with the proportion of 7.07 for males and 12.00 for females.

As shown in the Supplementary file Figure 1, it is clear that for secondhand smoke, the ASMR and ASDR for women in the three age groups are higher than those for men. In contrast, for active smoking and tobacco use, the rates for men are significantly higher than those for women. Additionally, the trends for both males and females are similar, with no significant differences.

In line with the above results, individuals aged ≥70 years exhibit elevated mortality and DALY rates, due to both active smoking and secondhand smoke-related COPD. The number of death cases and DALY cases attributed to active smoking and secondhand smoke exposure among individuals aged ≥70 years is also the highest (Supplementary file Figure 2).

**Figure 2:**
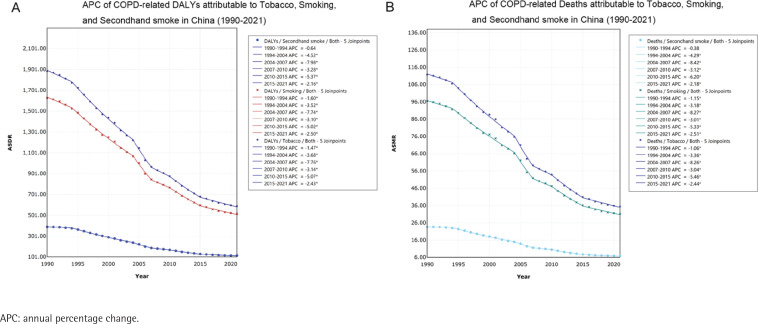
APC of COPD attributed to tobacco in China (1990–2021): A) APC of COPD-related DALYs attributable to tobacco use in China; B) APC of COPD-related deaths attributable to tobacco use in China

From 1990 to 2021, China’s overall APC values were below zero (Figure 2). This highlights a general declining trend in burden of COPD caused by tobacco use during this period. In the Supplementary file, for active smoking as well as secondhand smoking, it can be seen that, APC for both males and females was overall negative. This means that, whether from active smoking or secondhand smoke, the ASMR and ASDR for both males and females have been on a declining trend from 1990 to 2021. Meanwhile, this can manifest in any age group.

## DISCUSSION

This study utilizes data from the Global Burden of Disease 2021 to investigate the burden of COPD attributable to tobacco use among individuals aged ≥15 years in China from 1990 to 2021. It analyzes trends over this period and aims to offer relevant insights. The findings of this study reveal that, in 2021, both the age-standardized mortality rate and age-standardized DALY rate for COPD attributable to tobacco in China were higher than those observed globally and across various SDI regions. This indicates that China continues to bear one of the heaviest burdens of tobacco-related COPD in the world. COPD remains a significant public health concern, especially in countries with lower socio-economic levels^17^. This disparity is not only apparent on an international scale; significant socio-economic gaps exist within China as well. Individuals with lower socio-economic status in Chinese society are at a higher risk of developing COPD, a situation likely linked to insufficient tobacco control measures and unequal access to healthcare services in their regions. The imbalanced distribution of healthcare services in China represents a significant public health challenge, further intensified by the growing regional economic disparities^18^. These findings indicate that China must enhance its tobacco control efforts. Urgent and effective anti-smoking measures should be implemented, alongside initiatives to promote smoke-free environments, with a particular focus on supporting individuals from lower economic backgrounds.

The research findings indicate that from 1990 to 2021, the burden of COPD attributed to tobacco in China has demonstrated an overall declining trend. Both ASDR and ASMR have decreased, with AAPC<0. China’s rate of decline is markedly greater than the global average; however, the ASMR still exceeds the global average. This implies that China is achieving meaningful headway in preventing and managing COPD linked to tobacco use. Nevertheless, the overall burden of COPD remains substantial and surpasses the global average. This situation may be closely linked to factors such as China’s large population, an aging demographic, high smoking rates, low rates of pulmonary function testing, and inadequate diagnosis and treatment^19^.

Our study reveals that tobacco-related COPD is significantly affected by age and gender. The COPD mortality rate and DALY rate attributed to tobacco in China exhibit significant gender differences, with women experiencing notably lower rates compared to men. This variation could be related to the considerably elevated smoking rates observed in men. Additionally, women tend to be more receptive to health education, suggesting that they possess greater health awareness and demonstrate better adherence to medical advice than men^20^. This notable disparity underscores the need for gender-specific public health interventions. The increasing smoking rate among men, coupled with its direct correlation to the incidence of COPD, emphasizes the urgent requirement for smoking cessation programs tailored for men. Conversely, regarding secondhand smoke, we observe that the prevalence of COPD is marginally greater among women compared to men, potentially linked to women’s heightened vulnerability to the disease^21^.

The burden of COPD attributed to tobacco in China shows age-related differences, which is generally consistent with previous research findings^22,23^. The disease burden increases gradually with age, with both mortality and DALY rates rising progressively as age increases, peaking in the population aged ≥70 years. The study found that the mortality rate follows an almost exponential growth pattern with age, with mortality rates in the elderly significantly higher than in younger populations. This may be attributed to China’s current aging population, where older adults often have multiple underlying health conditions, lower immune function, and are more prone to adverse outcomes such as death, especially after alcohol consumption^24,25^. As a modifiable risk factor for COPD, tobacco has harmful health effects that are both chronic and delayed, directly related to how much and how long a person has smoked. As the smoking index increases, the detrimental impact of tobacco on health becomes more pronounced. Quitting smoking can reduce the risk of developing COPD among smokers, and the degree of risk reduction is dependent on the length of time since quitting. The longer the period of abstinence, the greater the reduction in the risk of COPD^26^. Compared to continuing smoking, quitting not only slows the decline in lung function but also improves survival rates, and this applies equally to patients with severe COPD^27^. In fact, the absolute risk of developing COPD does not decrease immediately after quitting smoking. Studies have demonstrated that while quitting smoking can lower overall mortality and mortality related to specific major causes, the reduction in COPD risk occurs over a period of years and follows a certain half-life^28^. About 20% of young smokers are expected to develop COPD by the time they reach the age of 40 years^29^. If health interventions are implemented early, particularly targeting male smokers, the risks associated with continued smoking can be significantly mitigated, greatly reducing the disease burden caused by tobacco. Older adults and men are most affected by the disease burden. In the future, it is recommended that more focus be placed on primary prevention of risk factors and targeted screening for these high-risk groups to further reduce the disease and economic burden attributable to tobacco-related COPD. Additionally, considering the significant wealth disparity in China, raising the price of cigarettes could help alleviate the burden of COPD and extend life expectancy, a point that has already been proven^30^.

Tobacco is a major contributor to the development of COPD, with both direct smoking and secondhand smoke significantly increasing the disease’s incidence. Both active and passive smoking raise the risk of COPD, which is consistent with our research findings. However, active smoking significantly increases the risk of developing chronic obstructive pulmonary disease, while non-smokers face a comparatively lower threat of severe acute exacerbations and mortality^31^. As the average daily smoking rate, age of initiation, and depth of inhalation increase, the risk of developing COPD correspondingly rises. For smokers, the most effective way to mitigate the harms of smoking is to quit as early as possible.

China continues to have the highest proportion of smokers in the world. However, in recent years, sustained economic growth, heightened public health awareness, and increased dissemination of information regarding the dangers of tobacco have led to a significant decline in smoking in public places. Consequently, the burden of COPD associated with tobacco use has decreased^32,33^. Prevention remains the most effective strategy for addressing COPD^3^. Furthermore, China has implemented relevant regulations and established a relatively comprehensive basic medical insurance system to ensure that patients receive appropriate treatment^34^.

### Limitations

Although our study is innovative and provides valuable insights, our study has certain limitations. Our study is based on the GBD database; therefore, residual confounding factors cannot be avoided. Joinpoint regression analysis methods can only describe the trend of changes in COPD mortality rates and cannot explain the associated factors. The results may lack external validation, especially when applied to different populations or regions, and may not be directly generalizable. The identified change points may not necessarily have biological or clinical significance, and further analysis may be needed to confirm the causes and impacts of these changes. Due to the reliance on data from the Global Burden of Disease Database, we lack specific data from most countries, including individual provinces and regions within China. Additionally, given China’s vast size, ethnic diversity, significant wealth disparity, and varying regional dietary habits, we were unable to conduct more detailed analyses or predict changes in the COPD disease burden attributable to tobacco in China over the next decade. The scope of our discussion does not include smokeless tobacco, electronic cigarettes (commonly referred to as e-cigarette products), and heated tobacco products. Finally, the potential interactions between tobacco use and other factors were not considered.

## CONCLUSIONS

The results of this study show that from 1990 to 2021, the COPD disease burden attributable to tobacco in the Chinese population has shown a declining trend, with significant gender and age differences. A large proportion of COPD disease burden is attributed to middle-aged and elderly men. This study is crucial for informing policy decisions and optimizing healthcare resource allocation. It may provide policymakers with new insights into the disparities in COPD disease burden, enabling them to make better policy decisions and allocate appropriate resources for COPD prevention.

## Supplementary Material



## Data Availability

The data supporting this research are available from the authors on reasonable request.
