# Supplementary material for: Chronic obstructive pulmonary disease burden attributable to tobacco and the trend change from 1990 to 2021 in China
Source: Tob Induc Dis. 2025 Feb 10;23:10.18332/tid/200196. doi: 10.18332/tid/200196 (PMC11808299; doi:10.18332/tid/200196)
Supplement: Supplementary file 1 [file TID-23-14-s1.pdf]

Supplementary file

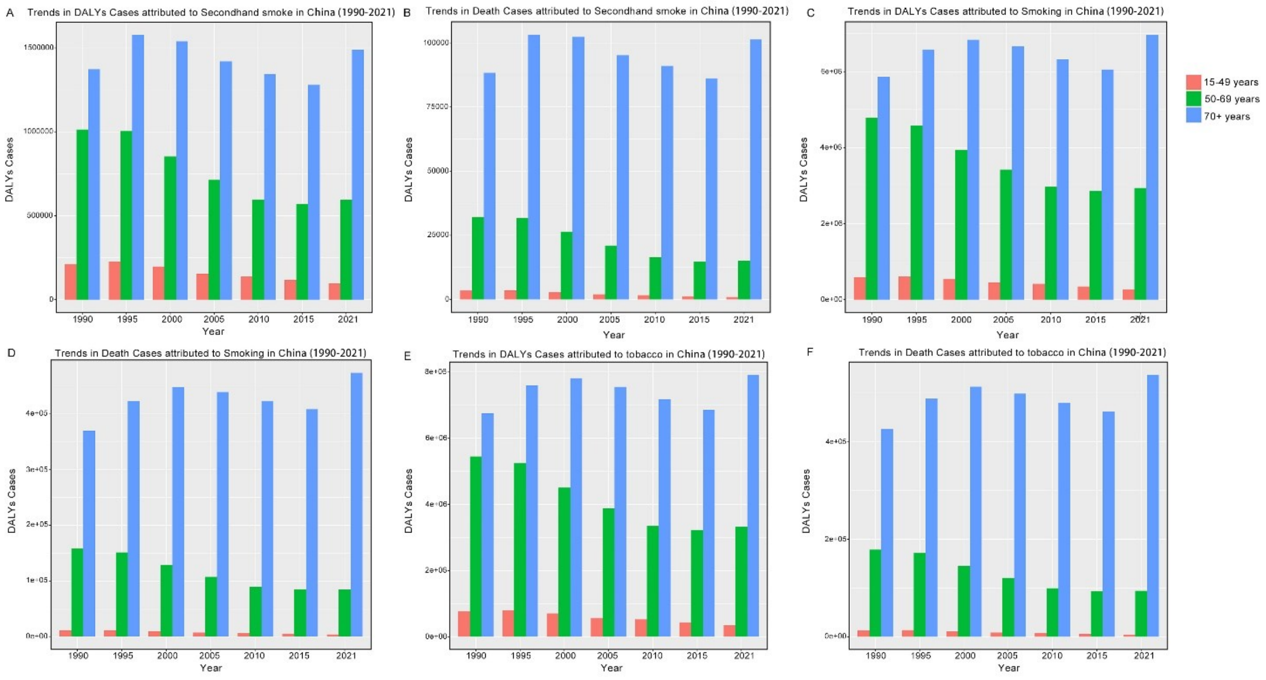

Supplementary file Figure 1. The disability-adjusted life years (DALY) and mortality rates of COPD attributed to Tobacco in different age groups in China: A) Trends in DALYs Rate attributed to Secondhand smoke in China; B) Trends in Death Rate attributed to Secondhand smoke in China；C) Trends in DALYs Rate attributed to Smoking in China; D) Trends in Death Rate attributed to Smoking in China; E) Trends in DALYs Rate attributed to Tobacco in China; F) Trends in Death Rate attributed to Tobacco in China. Smoking and secondhand smoke are subcategories of tobacco.

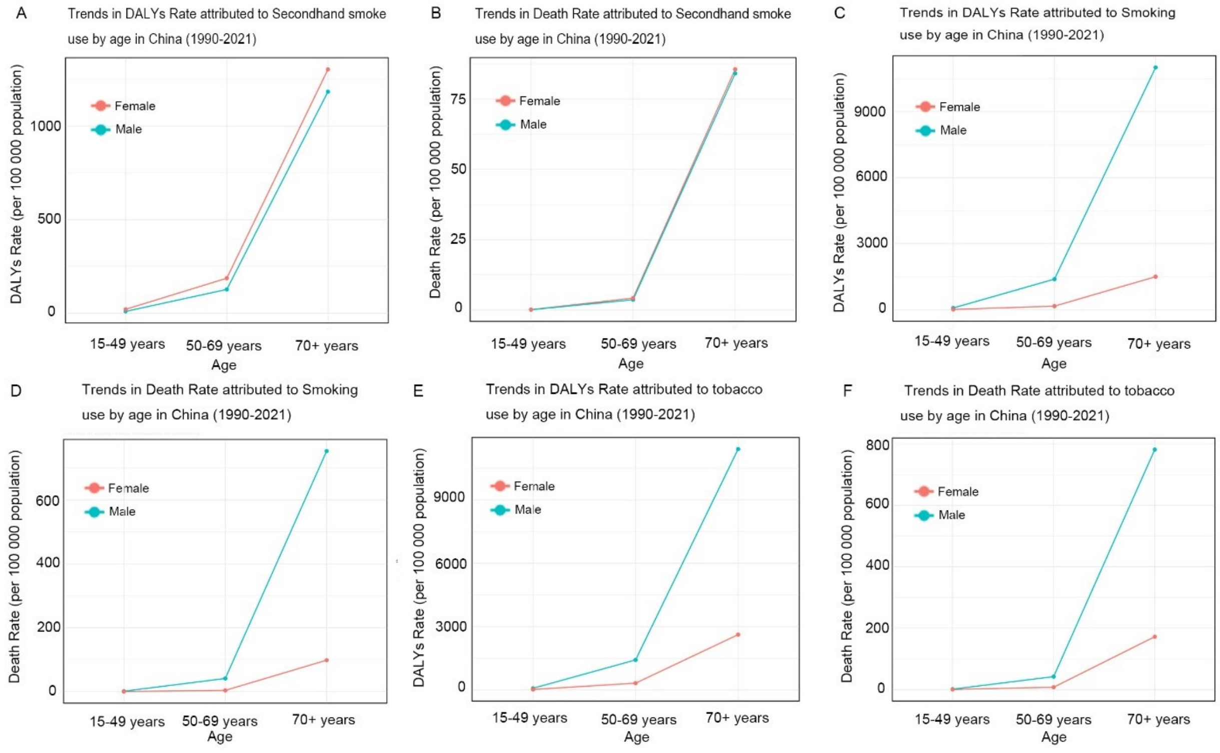

Supplementary file Figure 2. The DALYs and Death Cases of COPD attributed to Tobacco in different Years in China: A) Trends in DALYs Cases attributed to Secondhand smoke in China; B) Trends in Death Cases attributed to Secondhand smoke in China； C) Trends in DALYs Cases attributed to Smoking in China; D) Trends in Death Cases attributed to Smoking in China; E) Trends in DALYs Cases attributed to Tobacco in China; F) Trends in Death Cases attributed to Tobacco in China. Smoking and secondhand smoke are subcategories of tobacco.

Other supplementary documents.

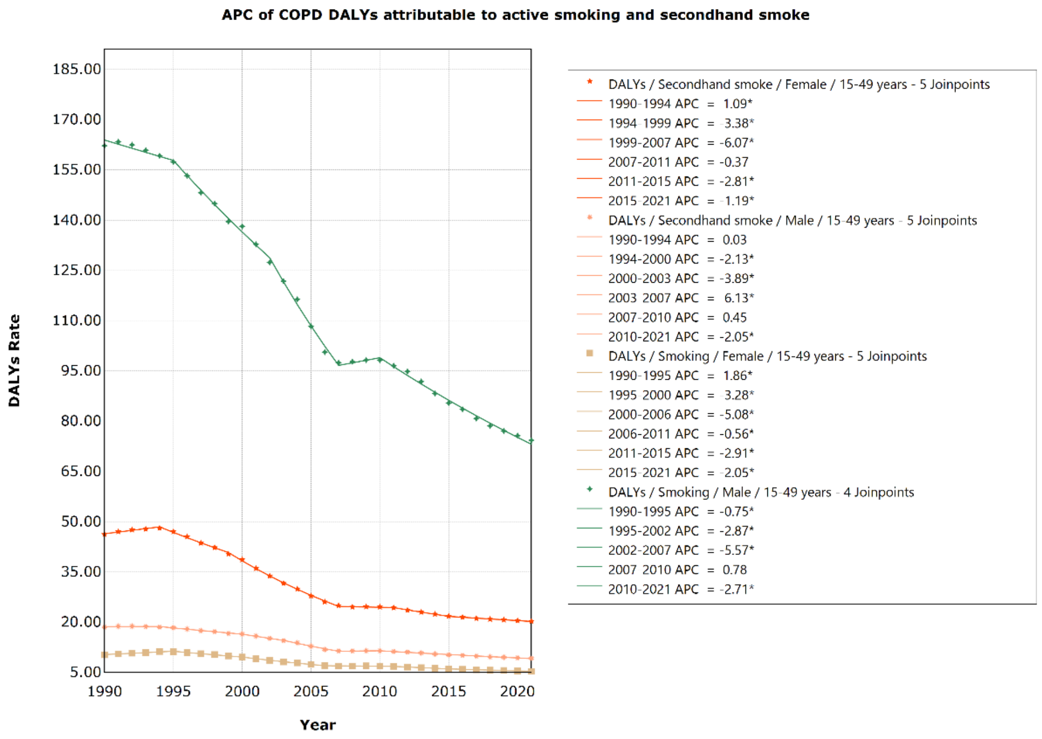

APC of COPD DALYs attributable to active smoking and secondhand use by 15-49 years in China（1990-2021）. Smoking and secondhand smoke are subcategories of tobacco.

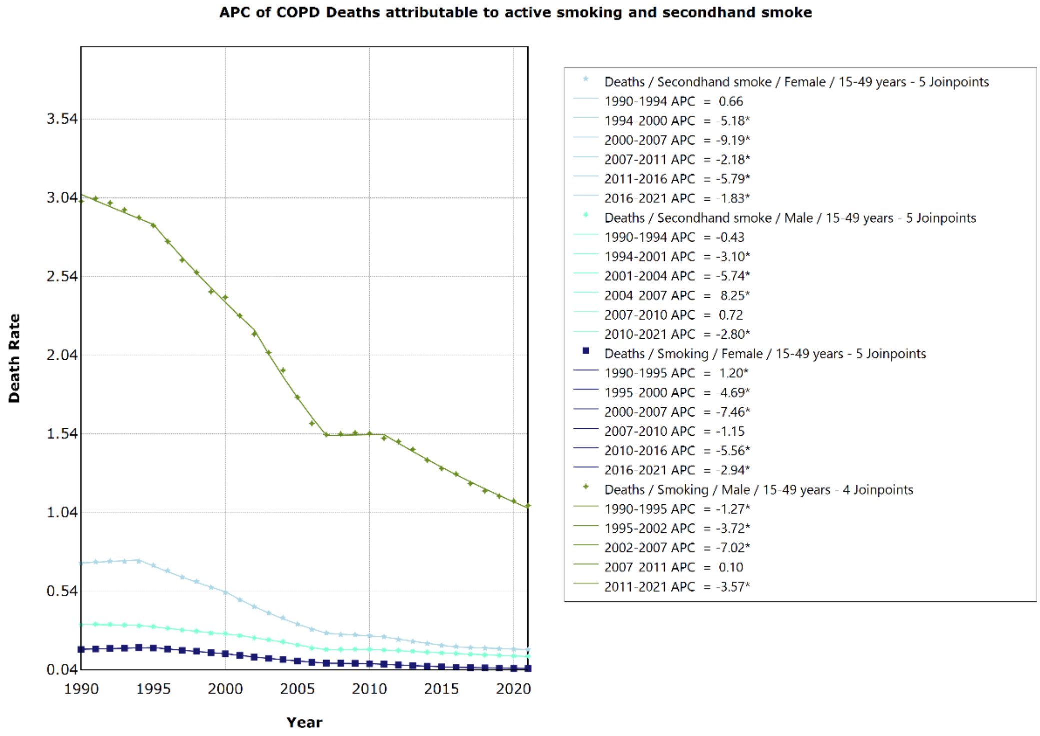

APC of COPD Deaths attributable to active smoking and secondhand use by 15-49 years in China（1990-2021）. Smoking and secondhand smoke are subcategories of tobacco.

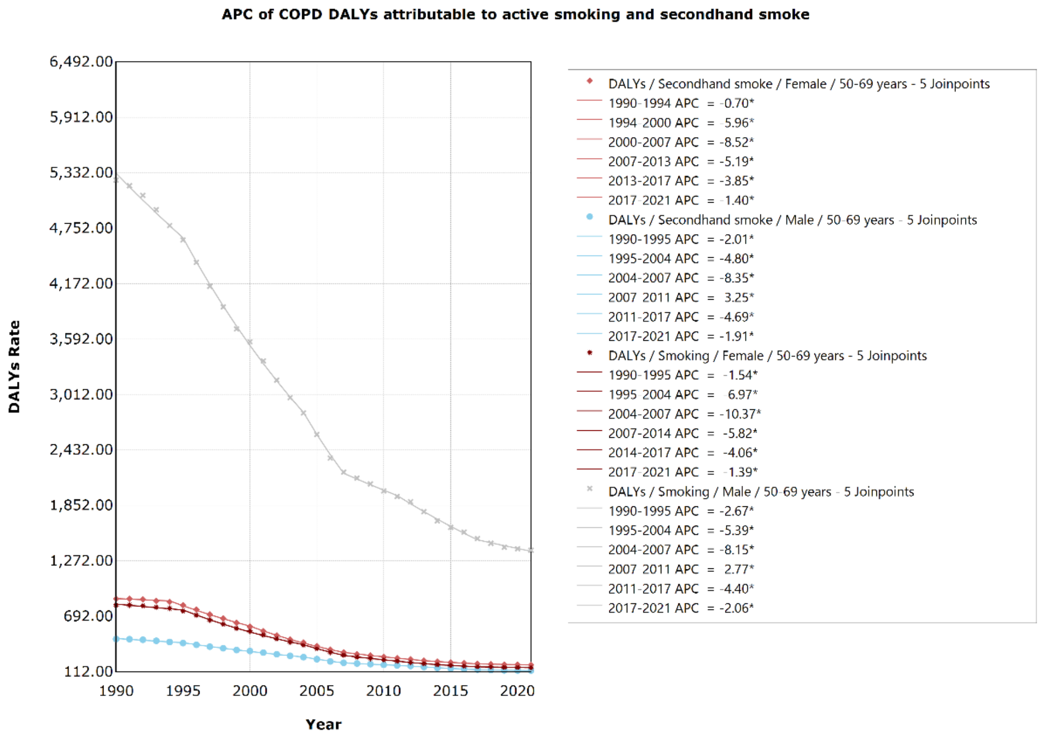

APC of COPD DALYs attributable to active smoking and secondhand use by 50-69 years in China（1990-2021）.Smoking and secondhand smoke are subcategories of tobacco.

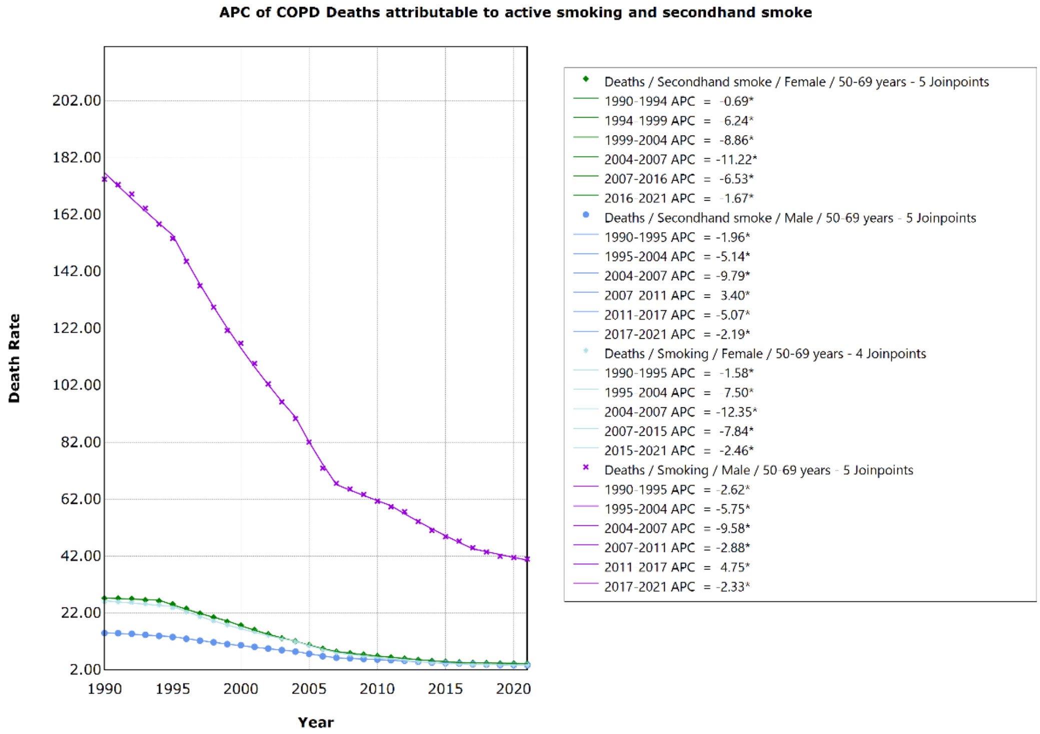

APC of COPD Deaths attributable to active smoking and secondhand use by 50-69 years in China（1990-2021）. Smoking and secondhand smoke are subcategories of tobacco.

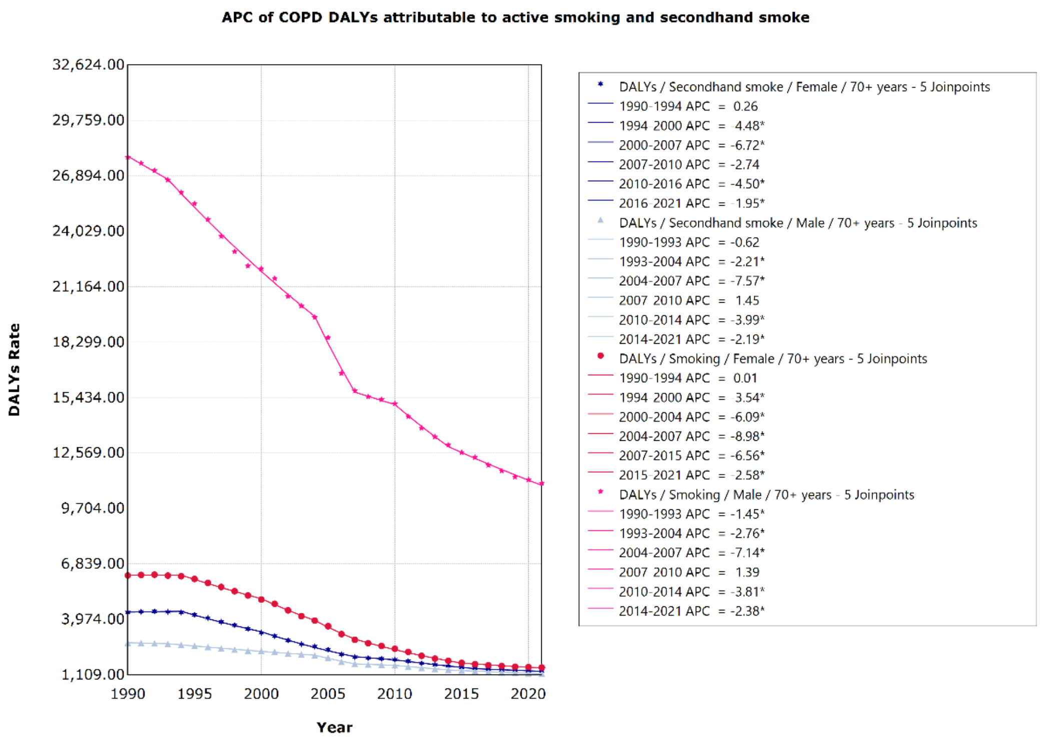

APC of COPD DALYs attributable to active smoking and secondhand use by 70+ years in China （1990-2021）. Smoking and secondhand smoke are subcategories of tobacco.

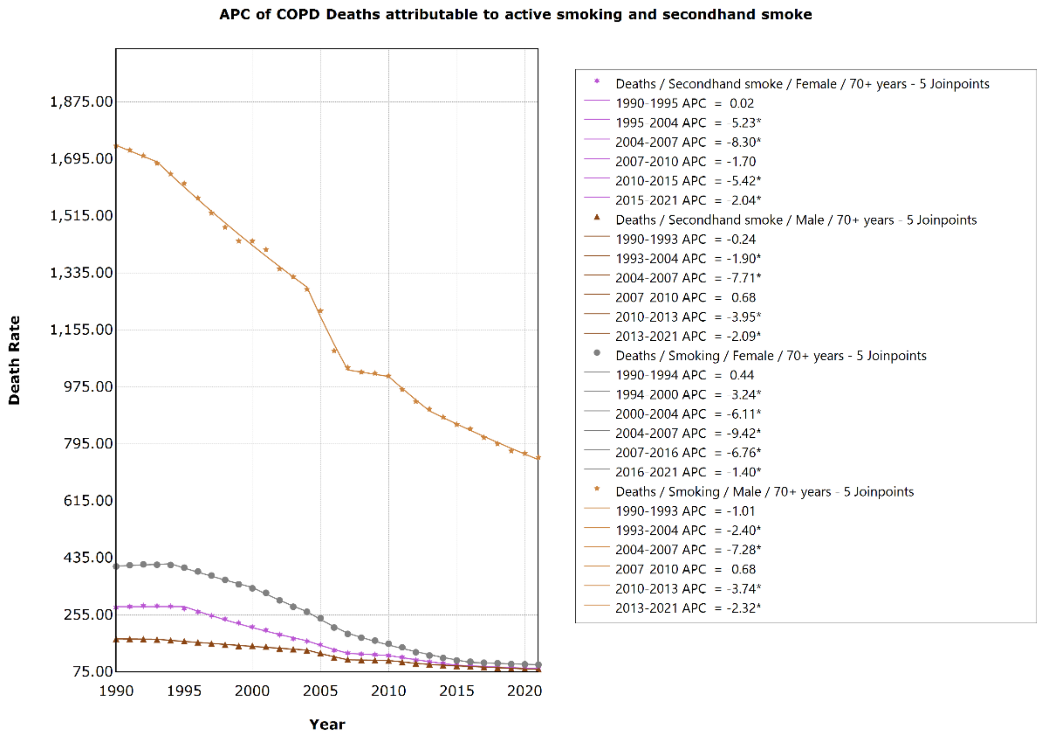

APC of COPD Deaths attributable to active smoking and secondhand use by 70+ years in China （1990-2021）. Smoking and secondhand smoke are subcategories of tobacco.v

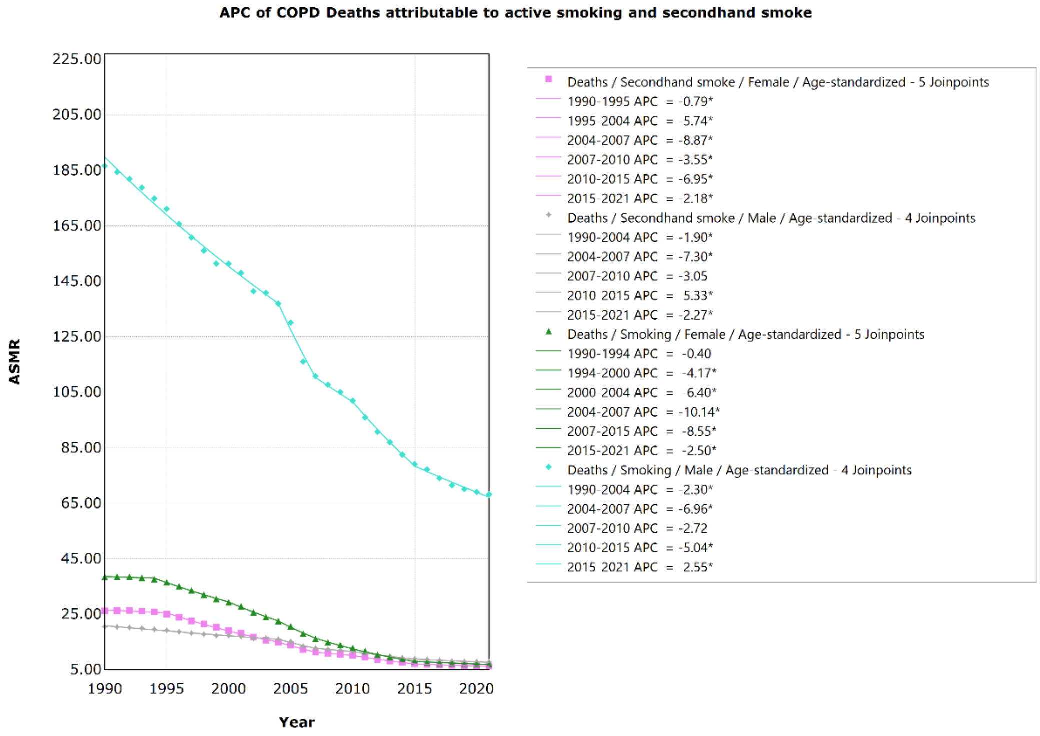

APC of COPD Deaths attributable to active smoking and secondhand use by Age-standardized in China（1990-2021）. Smoking and secondhand smoke are subcategories of tobacco.

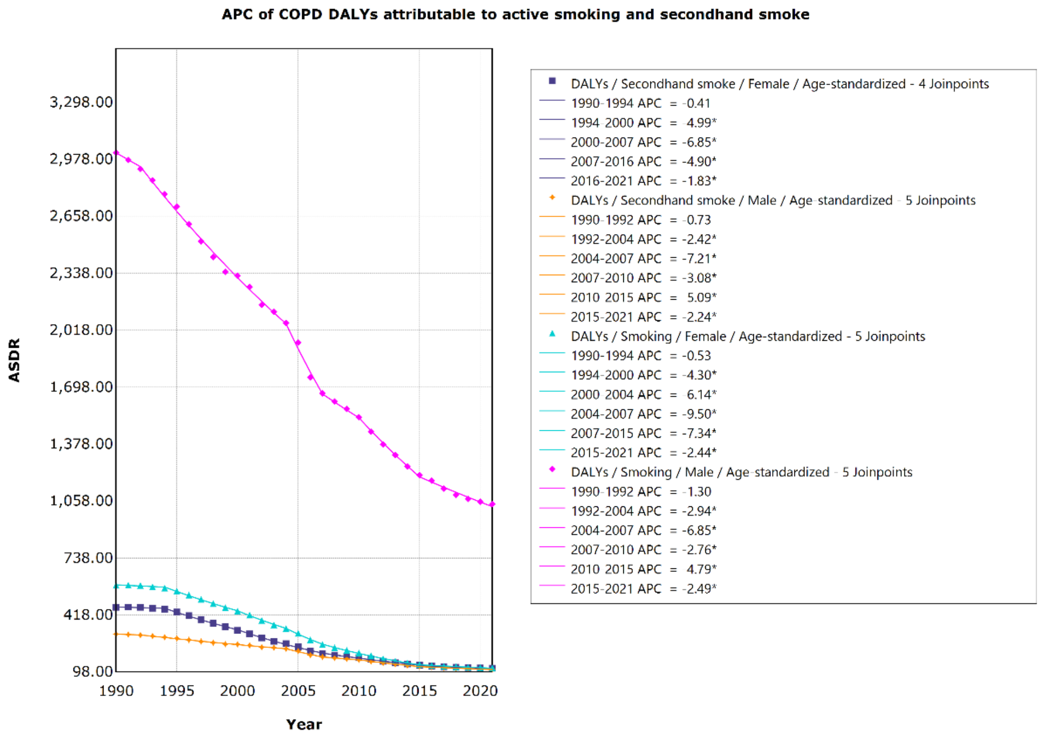

APC of COPD DALYs attributable to active smoking and secondhand use by Age-standardized in China （1990-2021）. Smoking and secondhand smoke are subcategories of tobacco.

© 2025 Zhang Z. et al.
